# Help seeking and mental health outcomes among South Asian young adult survivors of sexual violence in the New York State Region

**DOI:** 10.1186/s12889-022-13489-y

**Published:** 2022-06-08

**Authors:** Sahnah Lim, Shahmir H. Ali, Sadia Mohaimin, Ritu Dhar, Moitrayee Dhar, Farzana Rahman, Liza Roychowdhury, Tanzeela Islam, Nadia Islam

**Affiliations:** 1 Department of Population Health, Grossman School of Medicine, New York University, New York, NY USA; 2 Department of Social and Behavioral Sciences, School of Global Public Health, New York University, 708 Broadway, New York, NY 10003 USA

**Keywords:** Child sexual violence, Sexual assault, Stigma, Community-based participatory research

## Abstract

**Background:**

Sexual violence is a growing issue faced across diverse South Asian American communities under the backdrop of a distinct religious and cultural environment that intersects with the ability to prevent and manage this public health crisis. There is also growing attention on sexual violence experienced by younger or second-generation South Asian Americans, although little is known on the prevalence of this violence and its impact on health outcomes. Using data from a community-driven sexual violence survey, this study describes the experience of sexual violence and related help seeking behaviors and mental health outcomes among 18–34-year-old South Asian Americans living near the New York (NY) State region.

**Methods:**

Participants were recruited via social media to participate in an anonymous survey developed in partnership with an advisory board of South Asian young adult representatives. Data was analyzed descriptively and through adjusted logistic regression models.

**Results:**

Overall, responses from 335 sexual assault survivors were analyzed. Types of assault experienced included no-contact (97.6%), contact (75.2%), rape attempts (50.2%), rape (44.6%), and multiple rape (19.6%). Many reported perpetrators were South Asian (65.1%) or family members (25.1%). Only 27.6% indicated they had reported assaults to authorities or received services. In adjusted analyses, odds of help seeking were higher among participants who were older (AOR:1.10, 95%CI:1.02–1.20), were a sexual minority (lesbian, gay, bisexual) (1.98, 1.05–3.71), had a family member as the perpetrator (1.85, 1.01–3.40), had lower disclosure stigma (1.66, 1.16–2.44), and experienced depression (2.16, 1.10–4.47). Odds of depression were higher among sexual minority participants and lower among those with higher sexual assault disclosure stigma (3.27, 1.61–7.16; 0.68, 0.50–0.93).

**Conclusions:**

Findings call for greater targeted policy interventions to address the prevention of sexual violence among young South Asian Americans and greater focus on improving help seeking behaviors and improving mental health outcomes among survivors.

**Supplementary Information:**

The online version contains supplementary material available at 10.1186/s12889-022-13489-y.

## Background

Sexual violence has been identified as a significant public health challenge faced by Asian Americans [1]. The National Sexual Violence Resource Center defines sexual violence as being forced or manipulated into unwanted sexual activity without their consent and can include rape, sexual assault, unwanted sexual contact, and sexual harassment [2]. Asian Americans are now the fastest growing racial/ethnic group in the United States [3]. Overall, 19.6% of Asian or Pacific Islander women in the United States have reported lifetime experiences of rape, physical violence, or stalking by an intimate partner. While this prevalence is lower than other racial and ethnic subgroups [4], many specific Asian American subgroups (including South Asians) experience a disproportionate impact of gender-based violence compared to Asian American and US population averages [1]. There has been no nationwide surveillance of sexual assault among South Asian Americans. A number of community-based studies shows that the proportion of South Asian women who report experiencing some form of intimate partner violence has ranged from 21.2% to 40.8% [5–8], with most of these studies measuring physical intimate partner violence only. One study reported child sex abuse among 25.2% of South Asian adults participating in a web-based survey [9], and marital rape and issues of sexual control have been identified in South Asian immigrant communities [10]. Preliminary disaggregated analyses of gender-based violence indicators also show the prevalence of partner abuse among Asian Indians (19.5%) to be higher than Japanese (9.7%) and Chinese (9.7%) Americans [11]. Compared to African American and Hispanic survivors of domestic violence, South Asian survivors reported a significantly higher prevalence of being burned or scalded (68.4% compared to 22.2% and 13.6%, respectively) [12]. Reports of intimate partner emotional or psychological abuse among South Asians also vary but have been reported as consistently high, ranging from 35.8% among South Asians in one study to as high as 75% in particular subgroups [6, 13].

There is significant religious, cultural, and linguistic diversity within the South Asian community [14]. A majority of South Asian Americans trace their ethnic origins from India, Pakistan or Bangladesh, and (unlike many other Asian American subgroups) Hinduism and Islam represent the dominant religions practiced across the diaspora [15, 16]; such differences are notable given the complex role of religious and cultural beliefs on sex related stigma and attitudes [17]. These unique influences, coupled with the fact that South Asians are often overlooked in the contextualization of Asian Americans experiences, targeted research on South Asian community needs is warranted [18].

Moreover, South Asian young adults of immigrant families face a unique set of contextual factors related to the experience and impact of sexual violence, including living arrangements that frequently include parents or family members, acculturative stress, and navigating intergenerational cultural conflict [19, 20]. In particular, the individualistic values of American society can contrast with the collectivist, family-oriented values of Asian cultures. A meta-analysis among Asian and Latino samples demonstrated that acculturation mismatch was positively correlated with intergenerational cultural conflict, which in turn was correlated with adverse mental health outcomes [19]. The same study found that this relationship was stronger among young adults compared to adolescents. As such, there has been a growing call to better understand and intervene in the health needs of young adult children of Asian immigrant families [20], which likely have distinct research, program, and policy implications with respect to addressing sexual assault related concerns.

Sexual assault has been associated with a wide range of health indicators, particularly mental health outcomes such as depression and post-traumatic stress disorder (PTSD). In a meta-analysis on the mental health impact of intimate partner violence, women exposed to intimate partner violence were 2–3 times at risk for a major depressive disorder and 1.5–2 times at risk for elevated depressive symptoms and postpartum depression [21]. A further meta-analysis also observed higher odds of lifetime diagnosis of anxiety disorders, depression, eating disorders, PTSD, sleep disorders, and suicide attempts among those who had experienced sexual abuse [22]. Although preliminary evidence has similarly indicated that South Asian American survivors of intimate partner violence are more likely to report depression (31.8% vs. 10.2%) and anxiety (34.1% vs. 20.1%) [5], there is limited evidence on mental health outcomes among South Asian Americans, including the association between specific socio-demographic characteristics, stigma, and sexual assault perpetration related indicators and mental health outcomes [1]. Additionally, there have been recent calls to pay greater attention to the role of intersectional stress and trauma among sexual and gender minority Asian Americans (who may experience unique interpersonal discrimination, poor social support, internalized minority stress, and structural oppression), which may also contribute to disproportionate mental health burden among sexual and gender minority sexual violence survivors [23].

Seeking help or services related to sexual assault are also crucial in both the prevention of sexual violence and mitigating its health impact. However, issues related to shame, fear, lack of knowledge on resources, and interpersonal or community obstacles have been reported as key barriers to sexual assault help-seeking among Asian Americans [1, 24, 25]. Lack of culturally appropriate prevention strategies and resources specifically for South Asian individuals has also been identified as a barrier to seeking behaviors [26]. Among Indian and Pakistan American women, younger women were more likely to contact outside agencies (e.g., police, legal services, and domestic violence programs) regarding an assault, while second and 1.5 generation women were more likely to seek healthcare [27]. What remains unknown is whether help-seeking behaviors among young South Asian American sexual assault survivors may also be tied to perpetrator identity (e.g., South Asian or family status), the location of perpetration (e.g., at home), and the role of stigma when disclosing violence victimization to others (i.e., disclosure stigma), which may also be salient given the role of interpersonal and stigmatization related barriers observed in sexual assault help-seeking in other Asian American contexts [1, 24, 25].

Therefore, the aim of this study is to describe the experiences of young South Asian American sexual assault survivors and identify factors associated with both sexual assault help-seeking behaviors as well as mental health outcomes. The research questions of this current study are to 1) assess factors associated with sexual assault help-seeking (defined as seeking help from authorities and/or receiving services related to sexual assault), and; 2) assess factors associated with moderate to severe depression. By identifying salient factors contributing to health- and healthcare utilization-related disparities experienced by young South Asian American sexual assault survivors, findings have the potential to improve understanding on how policy makers and public health practitioners can better target sexual assault prevention interventions and better intervene in the needs of South Asian sexual assault survivors.

## Methods

### Data collection

This study was informed by principles of community-based participatory research (CBPR) [28]. At the start of the COVID-19 pandemic in 2020, there was a dramatic rise in disclosures of sexual assault by young South Asian women on social media accounts (Instagram) in the New York City region. A group of six young South Asian women came together to respond to these disclosures by partnering with an investigator at NYU Grossman School of Medicine that led to the current study. This coalition later became more formalized as RAISSE (Research & Action Improving South-Asian Sexual Education) and was involved in the study development, implementation, and analysis, and dissemination of results. For example, RAISSE members were involved in both the selection, adaptation, and development of measure for the survey, recruitment of participants via social media platforms, and interpretation of results. For dissemination, RAISSE members participated in the writing and review of this current manuscript; RAISSE members also developed a community friendly report, community friendly infographics, and a website to house all this information that could be distributed back to the participants and other stakeholders via social media.

An anonymous, online, self-administered survey (via REDCap) was developed to assess a wide array of survey items related to sexual assault related attitudes, behaviors, and mental health. Eligibility was defined as being: 1) aged 18–34 years old, 2) currently living or staying in the state of New York, New Jersey, or Connecticut, and 3) self-identifying as South Asian. A convenience sample of participants was recruited via social media (including platforms such as Instagram and Facebook) given the popularity of such platforms among young adults and its role in the inception of the RAISSE advisory board, its ability to enhance respondent-driven sampling, as well as its ability to rapidly collect anonymized data [29, 30]. A list of local, culturally tailored sexual violence related resources was also provided at the end of the survey. Survey content and all study materials was approved by the NYU Grossman School of Medicine Institutional Review Board.

### Measures

Socio-demographic survey items included age, sex at birth, gender identity, sexual orientation, country of birth, South Asian ethnic group, religion, and primary language spoken at home. Past sexual violence experiences were assessed using items adapted from the Sexual Experiences Survey Long Form Victimization (SES-LFV), which included both childhood and adulthood sexual violence experiences [31, 32] and has established validity and reliability [33, 34]. Adapted SES-LFV survey items were used to identify specific categories of sexual assault experiences; no contact (nine items, e.g., “someone made teasing comments of a sexual nature,” “someone showed me pornographic pictures”), contact, rape attempt, rape, and multiple rape (three or more rape experiences). Mental health outcomes included PTSD, measured through the 20-item PTSD Checklist for Diagnostic and Statistical Manual of Mental Disorders-Fifth Edition (PCL-5) [35], and depression, measured through the 10-item Centre for Epidemiologic Studies Depression Scale (CESD-10) [36]. PTSD was defined as exhibiting at least 1 symptom of Criteria B and C and 2 symptoms of Criteria D and E of the PCL-5 and has shown to be valid in South Asian samples [37]. Moderate to severe depression was defined as a CESD-10 score of 15 or higher; the CESD measure has shown to be valid among South Asian individuals [38]. Sexual violence-related and bystander attitude items were adapted from the revised Sexual Consent Scale [39], the Illinois Rape Myth Acceptance Scale Short Form [40], and the Bystander Scale [41]. Additional attitude items were developed based on consultations with the RAISSE advisory board (which were partly informed by conversations and anecdotes related to sexual violence among South Asian young adults being circulated on social media at the time). Attitude items comprised of Likert-type response options ranging from strongly agree to strongly disagree. Based on results of the exploratory factor analysis (see Analysis), we reverse coded the items (higher scores reflected greater sexual assault disclosure stigma), summed up the responses, and divided it by the total number of items to create an average score on a continuous scale. For survivors of sexual assault, an additional set of questions were asked on whether participants had ever disclosed experiences with others, sought help from authorities, or received services related to the assault (the latter two defined collectively as help seeking behaviors).

### Analysis

Analyses were conducted among participants who indicated experiences of any type of sexual violence (*n* = 335). Exploratory factor analyses were first conducted among sexual assault related attitude items to identify salient constructs in the data incorporating the aforementioned adapted survey items from past scales with the additional survey items developed by the RAISSE advisory board. While most sexual assault and bystander related attitude items did not coalesce around any singular factors, a 1-factor disclosure stigma scale was identified comprising of four items (Additional File 1) that were specifically related to perceived fear or difficulty of disclosing sexual assault due to cultural, religious, and/or familial concerns (e.g., “I am afraid that disclosing my sexual assault will bring shame and isolation to my family”); the model fit indices suggested a moderate fit to the data (RMSEA = 0.295; TLI = 0.774), and internal consistency of the scale was high (alpha = 0.88). Participant characteristics, select sexual assault experience and attitude items, and mental health outcomes were first descriptively analyzed. Multivariable logistic regression analyses were then conducted to assess factors associated with 1) sexual assault help-seeking, and 2) moderate or severe depression (with a supplemental analysis of PTSD). Variables included in the adjusted models included demographic variables (i.e., age, sex at birth, US-born status) and variables that displayed statistically significant differences in bivariate analyses of outcome measures. All analyses were conducted in R [42].

## Results

### Participant characteristics

A total of 393 participants completed the survey, of which 335 (85.2%) reported being a survivor of any kind of sexual assault (Table 1). The average age of survivors was 23.1 years old (SD: 3.6) and were majority female (89.5%), heterosexual (73.6%), and US-born (74.0%).

**Table 1:** Sociodemographic characteristics and sexual assault related outcomes among sample of young South Asian sexual assault survivors (*N* = 335)

	N or mean	% or SD
Age, mean (SD)	23.1	3.6
Sex at birth		
Female	298	89.5%
Male	35	10.5%
Sexual orientation		
Straight	242	73.6%
Lesbian, Gay, Bisexual (LGB) +	87	26.4%
Ethnicity		
Indian	101	30.3%
Bangladeshi	154	46.2%
Pakistani	36	10.8%
Other/Mixed South Asian	42	12.6%
Religion		
Muslim	162	49.1%
Hindu	63	19.1%
Christian	21	6.4%
Agnostic/Atheist/Non-Religious	34	10.3%
Other/Multiple Religious	50	15.2%
US-born		
Yes	248	74.0%
No	87	26.0%
Sexual violence—lifetime (including childhood)		
No contact (Yes)	324	97.6%
Contact (Yes)	248	75.2%
Rape Attempt (Yes)	165	50.2%
Rape (Yes)	146	44.6%
Multiple Rape (> 3 times) (Yes)	44	19.6%
Sexual violence—Childhood		
No contact (Yes)	291	89.8%
Contact (Yes)	176	54.0%
Rape Attempt (Yes)	106	33.3%
Rape (Yes)	78	24.2%
Multiple Rape (> 3 times) (Yes)	32	11.6%
Perpetrator South Asian status		
Not South Asian	116	34.9%
South Asian	216	65.1%
Most common type of perpetrator (not mutually exclusive)		
Family member	84	25.1%
Romantic/sexual partner	92	27.5%
Stranger	98	29.3%
Most common location of perpetration (not mutually exclusive)		
Home	107	31.9%
Home of a family/friend	101	30.1%
Public place	96	28.7%
Disclosure and help-seeking among victim/survivors (*N* = 286)		
Disclosed sexual violence to someone (Yes)	246	86.9%
Reported assault to authorities or received services (Yes)	79	27.6%
Disclosure stigma score, mean (SD) (N = 286)	2.01	0.95
Depressive symptoms		
None or mild	93	30.8%
Moderate to severe	209	69.2%
Post-traumatic stress disorder		
No PTSD	124	41.5%
PTSD	175	58.5%

A majority of sexual assault survivors had experienced no contact (97.6%) or contact (75.2%) assaults, followed by rape attempts (50.2%), rape (44.6%), and multiple rapes (19.6%). A large proportion of survivors reported that at least one of their perpetrators were South Asian (65.1%), with many reporting perpetrators to specifically be family members (25.1%), romantic or sexual partners (27.5%), and/or strangers (29.3%). The most common location of sexual assault perpetration was at home (31.9%) or home of a family or friend (30.1%; not mutually exclusive). While a majority of survivors reported disclosing their assault experience with someone (86.9%), only 27.6% indicated that they had reported the assault(s) to authorities or received services. Prevalence of mental health outcomes were high in the sample, with 69.2% displaying symptoms of moderate to severe depression and 58.5% reporting symptoms of PTSD.

### Help seeking behaviors

In adjusted analyses, the odds of seeking help (reporting the assault to authorities or receiving services) among sexual assault survivors (Table 2) were higher among older participants (AOR:1.10, 95%CI:1.02–1.20), those identifying as LGB + (AOR:1.98, 95%CI:1.05–3.71), and those whose perpetrator was a family member (AOR:1.85, 95%CI:1.01–3.40), while lower among those with higher sexual assault disclosure stigma (AOR:0.60, 95%CI:0.41–0.86), and those with moderate to severe depression (AOR:2.16, 95%CI:1.10–4.47). When looking only among survivors of either contact-based sexual assault or rape, the results remained similar; we saw an increased odds of seeking help associated with identifying as LGB + (AOR:2.09, 95%CI:1.09–4.01), and having moderate to severe depression (AOR:2.16, 95%CI:1.04–4.76) (not shown in tables), and lower odds among those with greater sexual assault disclosure stigma (AOR: 0.63, 95%CI: 0.42-0.91).

**Table 2:** Factors associated with increased odds of help seeking (i.e., reporting assault to authorities or receiving services) among South Asian sexual assault survivors, (*N* = 286)

		Total (n/mean)	Yes (n/mean)	Yes (%/SD)	UOR	AOR ^
Age, mean (SD)		23.0	23.6	3.49	1.06 (0.99–1.14)	**1.10 (1.02–1.20)
Sex at birth	Female	261	73	28.0%	Ref	Ref
	Male	23	6	26.1%	0.91 (0.32–2.28)	1.12 (0.36–3.15)
Sexual orientation	Straight	200	49	24.5%	Ref	Ref
	LGB +	81	29	35.8%	1.72 (0.98–2.99)	*1.98 (1.05–3.71)
Muslim	No	147	40	27.2%	Ref	Ref
	Yes	135	38	28.1%	1.05 (0.62–1.77)	1.10 (0.42–1.40)
Hindu	No	229	68	29.7%	Ref	Ref
	Yes	53	10	18.9%	0.55 (0.25–1.12)	0.80 (0.34–1.75)
US-born	No	76	23	30.3%	Ref	Ref
	Yes	210	56	26.7%	0.84 (0.47–1.51)	0.79 (0.42–1.50)
South Asian Perp	No	70	13	18.6%	Ref	Ref
	Yes	215	66	30.7%	1.94 (1.02–3.92)	1.05 (0.48–2.39)
Perp. Type	Not family member	202	46	22.8%	Ref	Ref
	Family member	84	33	39.3%	**2.19 (1.27–3.80)	*1.85 (1.01–3.40)
Perp. Location	Not home	179	43	24.0%	Ref	Ref
	Home	107	36	33.6%	1.60 (0.94–2.72)	0.89 (0.45–1.71)
Disclosure stigma		2.0	1.8	0.9	**0.65 (0.47–0.88)	**0.60 (0.41–0.86)
Depression status	None or mild	77	14	18.2%	Ref	Ref
	Moderate to severe	186	59	31.7%	*2.09 (1.11–4.16)	*2.16 (1.10–4.47)

^*^p < 0.05; **p < 0.01; ***p < 0.001; ^Adjusted for age, sex at birth, US-born, perp. type, disclosure stigma, depression status

### Depression and PTSD

In adjusted analyses, odds of the depression (Table 3) among sexual assault survivors were higher among LGB + participants (AOR:3.27, 95%CI:1.61–7.16) and lower among those with higher sexual assault disclosure stigma (AOR:0.68, 95%CI:0.50–0.93). Although odds of depression were also higher among those with a South Asian perpetrator and lower among older participants, when adjusted for other variables these relationships did not remain statistically significant. An additional analysis of correlates of PTSD (not displayed) were conducted with similar findings, with lower PTSD being experienced by those of an older age (AOR:0.89, 95%CI:0.82–0.96), males compared with females (AOR:0.31, 95%CI:0.82), and higher PTSD among participants who were second-generation (AOR:2.05, 95%CI:1.11–3.79), experienced sexual violence at home (AOR:1.98, 95%CI:1.07–3.72), and had higher sexual assault disclosure stigma (AOR:1.57, 95%CI:1.18–2.10).

**Table 3:** Factors associated with increased odds of depression among South Asian sexual assault survivors, (*N* = 302)

		Total (n/mean)	Yes (n/mean)	Yes (%/SD)	UOR	AOR^
Age, mean (SD)		23.1	22.7	3.49	*0.92 (0.86–0.99)	0.94 (0.87–1.01)
Sex at birth	Female	268	188	70.1%	Ref	Ref
	Male	32	19	59.4%	0.62 (0.30–1.35)	0.48 (0.18–1.27)
Sexual orientation	Straight	220	141	64.1%	Ref	Ref
	LGB +	78	66	84.6%	**3.08 (1.62–6.30)	**3.27 (1.61–7.16)
Muslim	No	155	102	65.8%	Ref	Ref
	Yes	143	104	72.7%	1.39 (0.85–2.28)	1.38 (0.76–2.49)
Hindu	No	240	170	70.8%	Ref	Ref
	Yes	58	36	62.1%	0.67 (0.37–1.24)	0.60 (0.30–1.22)
US-born	No	81	53	65.4%	Ref	Ref
	Yes	221	156	70.6%	1.27 (0.73–2.17)	1.30 (0.69–2.42)
South Asian perp	No	99	59	59.6%	Ref	Ref
	Yes	201	149	74.1%	*1.94 (1.16–3.24)	1.68 (0.86–3.25)
Perp. type	Not family member	222	150	67.6%	Ref	Ref
	Family member	80	59	73.8%	1.35 (0.77–2.43)	0.71 (0.34–1.49)
Perp. location	Not home	200	131	65.5%	Ref	Ref
	Home	102	78	76.5%	1.71 (1.01–2.99)	1.20 (0.63–2.30)
Disclosure stigma		2.0	1.9	0.9	**0.61 (0.46–0.81)	*0.68 (0.50–0.93)

^*^p < 0.05; **p < 0.01; ***p < 0.001; ^Adjusted for age, sex at birth, US-born, sexual orientation, South Asian perp., perp. location, disclosure stigma

## Discussion

Our study adds to the limited literature on data on sexual assault experiences and related outcomes among South Asian survivors of sexual violence. Study results showed that all types of sexual violence were common throughout the life course and that participants often experienced multiple forms of sexual violence and multiple instances of rape, which has been shown to worsen physical and mental health sequalae in other studies [43–45].

While disclosure of sexual assault experience was high, help-seeking in the form of reporting to authorities or receiving services was low. A nationally representative study conducted among women showed that White women were more likely to seek out services in their lifetime compared to Latina, Black, and Other race women [46]; unfortunately, there are no national racial/ethnic comparisons of help-seeking that present disaggregated data for Asian survivors, including South Asians. Help-seeking was associated with identifying as sexual minority, reporting a family perpetrator, lower sexual assault disclosure stigma, and depressive symptoms. Although temporality cannot be established from our cross-sectional study design, these associations point to the possibility that survivors who experience multiple oppression (i.e., sexual minority survivors), those who experience complex forms of sexual assault (i.e., victimized by a family member), or those who experience greater consequences of sexual assault (i.e., depressive symptoms [46]) are more compelled to seek out services. Additional outreach efforts are needed to increase overall help-seeking among all South Asian survivors, but also among those who are less likely to seek out services. Additional support may also be needed for those who already sought out services but could be experiencing worsening of well-being indicators such as depressive symptoms, particularly in the context of the stressors caused by the COVID-19 pandemic and recent rise in anti-Asian violence, which has not precluded South Asians [48, 49].

Sexual assault disclosure stigma related to religion, familial harmony, and other cultural factors unique to the South Asian context was associated with lower odds of help-seeking among our participants. Previous studies have documented how culturally-specific stigma is shaped by patriarchal norms and the importance of upholding family honor and reputation. In turn, stigma suppresses disclosure and help-seeking related to gender-based violence among South Asian survivors [50–52]. In particular, daughters are responsible for upholding their sexual purity in order to maintain family honor, which often intersects with Muslim, Hinduism, and other religion’s upholding of female virginity [53, 54]. More broadly, scholars have noted how conservative parental attitudes around sexual health (e.g., prohibition of premarital sex) of their adolescent and young adult children among South Asians can perpetuate heterosexism and/or sex-negativity, driven by British colonial ideals, as well as some mainstream doctrines of faiths practiced by South Asians (e.g. Islam and Hinduism) [17, 55].

Given the important role of community and family, it has been suggested that traditional psychological and feminist approaches to working with South Asian survivors of sexual assault are likely not appropriate or realistic [52]. For example, the Western emphasis on disclosure and speaking out are likely not manageable. Rather, explicitly tackling stigma through the lens of family, culture, and other gendered power structures is likely a more effective therapeutic approach. Further, family-based approaches and interventions at the structural and community-level to address stigma beyond the individual-level are needed. At the family-level, the common messaging for girls and women to “stay at home” must be recognized as particularly harmful since our study shows that violence most often occurs at the survivors’ own home and by a perpetrator who is a family member. Community-engaged (e.g., leveraging community health workers or peers and engaging community-based organizations) and faith-based approaches have been shown to be culturally appropriate and effective in addressing a wide variety of non-sexual health outcomes (e.g., chronic diseases) in the South Asian community and should be extended to address sexual violence [56, 57].

Further, future interventions should prioritize South Asian survivors who experience other forms of marginalization, including those with limited English proficiency, low socioeconomic status, and sexual and/or gender minority status. Moreover, internalized stigma represents a significant issue among LGB + South Asians and subsequently represents a significant help-seeking barrier [58]. Future research may benefit from measuring internalized stigma to assess its role in help seeking among LGB + South Asian survivors of sexual violence. Moreover, higher odds of depression among young LGB + South may be a reflection on the unique intersectional stress and trauma faced by the community in both in-group and out-group settings as both an ethnic and sexual minority [23]; further analysis on mental health and sexual violence outcomes among the LGB + participants in this sample have been describe elsewhere [59]. Thus, efforts may be needed to address the disproportionate mental health burden similarly experienced by LGB + South Asian survivors of sexual violence.

In addition, there exists a robust feminist anti-violence movement in South Asia, whose prior work and approaches should be leveraged. In South Asia, interventions aimed at reducing gender-based violence have focused on educational training to address bystander attitudes and behaviors related to sexual assault among South Asian men [60], while others have been connected with structural-level poverty-reduction, such as a state-sponsored intervention involving social action committees focused on social support building and para-legal training formed from self-help groups [61]. Moreover, efforts to reduce intimate partner violence in South Asia have also included collaboration with regional and municipal governments, non-governmental organizations, as well as more informal, localized governance infrastructure (such as religious organizations) to enact prevention policies and promotion campaigns [62].

There are a few limitations to the current study. While the objective the current study was not to assess prevalence of sexual violence among South Asian individuals, the non-random sampling method of the study limits generalizability of the study to other South Asian populations in the US. Future research is sorely needed to collect disaggregated, national-level data on sexual violence among South Asian individuals for equitable resource allocation, which would allow for cross racial/ethnic group comparisons. Our study sample included a small number of men and gender minority individuals preventing any meaningful sub-analyses for these groups; future studies should consider oversampling men as well as sexual and gender minority South Asian individuals. The help-seeking outcome variable groups both the act of reporting to authorities and receiving services together; receipt of social services that is often more survivor-friendly may be a substantively different experience from reporting to authorities. Disentangling the differences between these two help-seeking behaviors may be important for future studies. Lastly, while our study was able to recruit a relatively diverse sub-group of South Asian individuals, there are many other South Asian sub-groups that were not represented in our study, which in turn prevented a disaggregated analysis by South Asian sub-groups.

## Conclusions

Overall, our study demonstrates that sexual violence is a significant human right and public health issue among South Asian young adults and one that is interlaced with complex religious, cultural, and structural issues that are unique to this group. Our study was strengthened by a CBPR approach in which a South Asian young adult advisory board drove the research questions, study development, data collection, and interpretation of results. The study team and the RAISSE advisory board are planning to disseminate the study results via social media and other outlets (e.g., community forum) using community-friendly approaches, such as a community brief with infographics. Young adults are at a stage in their life course in which interventions could potentially have a long-lasting and strong impact [63]. Continued data collection to inform policy and development of culturally tailored interventions among this under-researched and underserved group is urgently needed.

## Supplementary Information

**Additional file 1.** 4-itemSouth Asian sexual assault disclosure stigma scale construct from exploratoryfactor analyses of sexual assault related attitudes.

## Data Availability

The datasets used and/or analyzed during the current study are available from the corresponding author on reasonable request.

## Abbreviations

AOR: Adjusted odds ratio
LGB +: Lesbian, gay, bisexual, and other sexual minorities
CBPR: Community-based participatory research
PTSD: Post-traumatic stress disorder
RAISSE: Reach & Action Improving South-Asian Sexual Education
SES-LFV: Sexual Experiences Survey Long Form Victimization
PCL-5: PTSD Checklist for Diagnostic and Statistical Manual of Mental Disorders Fifth Edition
CESD-10: 10-Item Centre for Epidemiologic Studies Depression Scale
