# Supplementary material for: Help seeking and mental health outcomes among South Asian young adult survivors of sexual violence in the New York State Region
Source: BMC Public Health. 2022 Jun 8;22:1147. doi: 10.1186/s12889-022-13489-y (PMC9174918; doi:10.1186/s12889-022-13489-y)
Supplement: Supplementary file 1 — Additional file 1. 4-itemSouth Asian sexual assault disclosure stigma scale construct from exploratoryfactor analyses of sexual assault related attitudes. [file 12889_2022_13489_MOESM1_ESM.docx]

Additional File 1: 4-item South Asian sexual assault disclosure stigma scale construct from exploratory factor analyses of sexual assault related attitudes

| Item | Loading |
| --- | --- |
| I am afraid that disclosing my sexual assault will bring shame and isolation to my family | 0.91 |
| I am afraid that disclosing my sexual assault will ruin my relationship with my parents and family members | 0.87 |
| I find it difficult to speak of my assault due to perceived stigma within South Asian culture | 0.75 |
| I find it difficult to speak of my assault due to perceived stigma within my religion | 0.69 |
